# Treatment of chronic migraine with transcutaneous stimulation of the auricular branch of the vagal nerve (auricular t-VNS): a randomized, monocentric clinical trial

**DOI:** 10.1186/s10194-015-0543-3

**Published:** 2015-07-09

**Authors:** Andreas Straube, J. Ellrich, O. Eren, B. Blum, R. Ruscheweyh

**Affiliations:** Klinik und Poliklinik für Neurologie, Oberbayerisches Kopfschmerzzentrum, Klinikum Großhadern, Ludwig-Maximilians-Universität München, Marchioninistr. 15, 81377 Munich, Germany; Department of Health Science and Technology, Professor Dr. med. Jens Ellrich, Aalborg University, Fredrik Bajers Vej 7D2, DK-9220 Aalborg, Denmark; Cerbomed GmbH, Medical Valley Center, Henkestr. 91, 91052 Erlangen, Germany

**Keywords:** Sensory nerve, Neuromodulation, Clinical study, Chronic headache, Electrical pulses

## Abstract

**Background:**

Aim of the study was assessment of efficacy and safety of transcutaneous stimulation of the auricular branch of the vagal nerve (t-VNS) in the treatment of chronic migraine.

**Methods:**

A monocentric, randomized, controlled, double-blind study was conducted. After one month of baseline, chronic migraine patients were randomized to receive 25 Hz or 1 Hz stimulation of the sensory vagal area at the left ear by a handhold battery driven stimulator for 4 h/day during 3 months. Headache days per 28 days were compared between baseline and the last month of treatment and the number of days with acute medication was recorded The Headache Impact Test (HIT-6) and the Migraine Disability Assessment (MIDAS) questionnaires were used to assess headache-related disability.

**Results:**

Of 46 randomized patients, 40 finished the study (per protocol). In the per protocol analysis, patients in the 1 Hz group had a significantly larger reduction in headache days per 28 days than patients in the 25 Hz group (−7.0 ± 4.6 vs. −3.3 ± 5.4 days, *p* = 0.035). 29.4 % of the patients in the 1 Hz group had a ≥50 % reduction in headache days vs. 13.3 % in the 25 Hz group. HIT-6 and MIDAS scores were significantly improved in both groups, without group differences. There were no serious treatment-related adverse events.

**Conclusion:**

Treatment of chronic migraine by t-VNS at 1 Hz was safe and effective. The mean reduction of headache days after 12 weeks of treatment exceeded that reported for other nerve stimulating procedures.

## Background

Migraine is a frequent neurological disorder. In some patients, episodic migraine (with < 15 headache days per month) evolves towards chronic migraine, which is characterized by ≥15 headache days per month of which ≥ 8 have migraine-like features [1], see also: http://ihs-classification.org/de/0_downloads/. Chronic migraine affects approximately 1.3 to 2.4 % of the general population [2]. It is associated with significant disability and reduced health-related quality of life and often complicated by overuse of acute pain medications [3, 4]. Up to now, randomized controlled trials showing a significant effect in the treatment specifically of chronic migraine have been published only for topiramate and onabotulinumtoxin A [5, 6]. Treatment of chronic migraine is often difficult, with significant numbers of patients not responding to pharmacological management.

In recent years, neuromodulation was introduced in the treatment of headache [7]. Invasive occipital nerve stimulation (ONS) has been investigated for the treatment of chronic migraine, with inconsistent results [8–10]. Significant reduction in headache days was demonstrated in only one of the three studies, which however did not meet its primary endpoint (a 50 % reduction of mean daily pain ratings) [10]. A major disadvantage of ONS is the safety profile with frequent adverse events such as infections, lead migration or lead disconnection [8, 10]. This is also the reason why in some health markets the reimbursement of ONS was stopped by the regulatory administration. Thus, less invasive forms of neuromodulation such as transcutaneous electrical nerve stimulation are under investigation. For example, supraorbital transcutaneous stimulation for 3 months has been shown to be effective for the preventive treatment of episodic migraine (active treatment: 38 % responders, sham: 12 % responders, p < 0.05) [11].

Vagal nerve stimulation using implanted electrodes is used as a treatment option in otherwise therapy-refractory epilepsy and depression [12]. Case reports and small series of patients who received an implanted vagal nerve stimulator for treatment of epilepsy and had comorbid migraine suggest that VNS may have a preventive effect in migraine [13–16]. A recently developed medical device (NEMOS®, cerbomed, Erlangen, Germany) allows for non-invasive, transcutaneous stimulation of the auricular branch of the vagus nerve (auricular t-VNS) using a special ear electrode. Auricular t-VNS excites thick myelinated sensory Aβ-fiber afferents in the vagal nerve, activating the nucleus of the solitary tract [17, 18]. Effects on autonomous activity have been demonstrated in healthy subjects where auricular t-VNS increases heart rate variability [19]. Anticonvulsive effects in rodents are similar to those achieved with invasive VNS [18]. Functional imaging during auricular t-VNS has shown a pattern consistent with afferent vagal stimulation [20, 21]. Both invasive VNS and auricular t-VNS reduce pinprick and pressure pain in humans [22, 23]. In addition, a recent observational study has suggested that t-VNS to the right cervical branch of the vagus nerve (cervical t-VNS) may be effective for acute migraine treatment [24]. In the present study, we investigated the effect of auricular t-VNS on chronic migraine.

## Methods

This was a monocentric, prospective, double-blind, randomized, parallel-group, controlled trial analyzed both on intention-to-treat basis (ITT), and on per protocol basis (PP). The trial was conducted in a German tertiary headache outpatient clinic (Department of Neurology, University of Munich). The study was approved by the ethics committee of the medical faculty of the University of Munich and written informed consent was obtained from all participants. The study is registered in the German Clinical Trials Register (DRKS00003681).

### Study participants

Men or women between 18 and 70 years with a diagnosis of chronic migraine according to the ICHD-IIR (code A1.5.1.) (http://ihs-classification.org/de/0_downloads/), duration of ≥ 6 months, no migraine-prophylactic medication or stable migraine-prophylactic medication for ≥1 month, and stable acute medication were eligible, medication overuse was not an exclusion criterion.

Patients were excluded if they suffered from other primary or secondary headaches, severe neurologic or psychiatric disorders including opioid- or tranquilizer-dependency, cranio-mandibulary dysfunction, fibromyalgia, had a Beck’s Depression Inventory (BDI [25]) score >25 at the screening visit, anatomic or pathologic changes at the left outer ear, currently participated in another clinical trial, or were unable to keep a headache diary. Pregnant or breast-feeding women were also excluded. A pregnancy test was performed at the screening visit in women of childbearing potential and they were required to use a reliable means of contraception. In addition, patients who had less than 15 headache days per 28 days during the 4-week baseline period were excluded.

### Study design (Fig. 1)

**Fig. 1:**
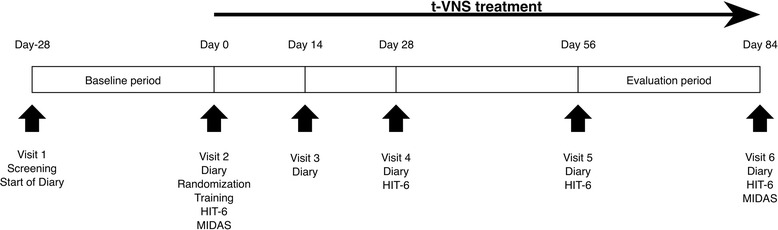
Study design

**Fig. 2:**
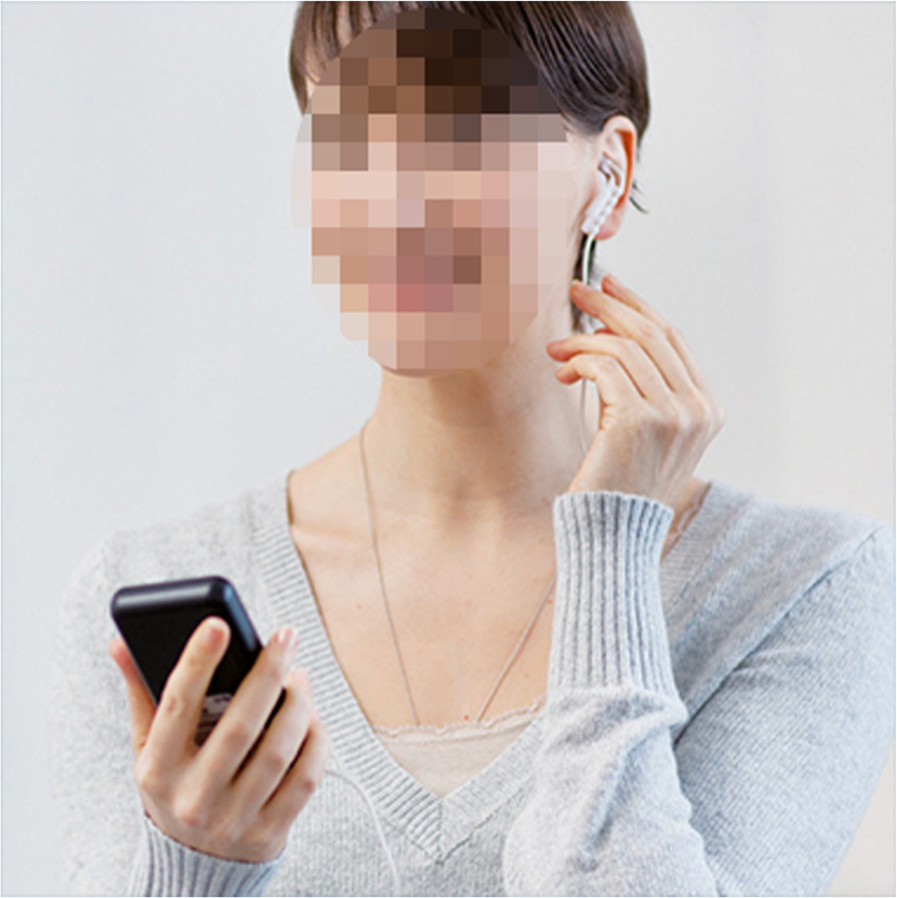
NEMOS® device and positioning of the electrode for stimulation of the vagus afferents at the concha

The study consisted of a 4-week screening period (“baseline”) followed by a 12-week randomized, double-blind, parallel-group treatment period with either 1 Hz or 25 Hz tVNS with the NEMOS® device (Fig. 2). Adverse events were recorded at visits 2 to 6. Compliance with stimulation was checked at visits 3 to 6 by reading out the NEMOS® device and quantified in percent of the intended daily stimulation time (4 h). Re-training was administered during visits 3 to 6 as necessary. The Migraine Disability Assessment (MIDAS [26]) and the Headache Impact Test (HIT-6 [27]) were filled in by the patient as indicated in Fig. 1. Patients kept a paper-and-pencil headache diary during the entire period, handing in their diaries and receiving a fresh sheet at each visit. In the diary, patients indicated for every day (1) headache duration in hours, (2) headache intensity (on a 0 to 10 numerical rating scale: 0, no pain; 10, strongest pain imaginable), and (3) intake of acute headache medication (analgesics, triptans).

Sample size calculations were based on published studies on successful pharmacological treatment of chronic migraine (mean effect size: −4,68 headache days/month after removal of the placebo effect) [5, 6, 28, 29]. To detect an effect of this size with an α error of 0.05 and a power of 0.80, a group size of 49 patients per treatment group was estimated, including 10 % drop-out. An interims analysis after 46 patients was planned. Since patient recruitment was slower than expected, the sponsor decided to terminate the study at the interims analysis, and no further patients were enrolled.

### Neurostimulation

The NEMOS® t-VNS device (Cerbomed, Erlangen, Germany) is a transcutaneous vagus nerve stimulator designed for electrical stimulation at the concha of the outer ear, which receives sensory innervation from the auricular branch of the vagal nerve (Fig. 2). The NEMOS® device has received the CE mark for treatment of pain (CE0408) and is registered in the European Databank on Medical Devices (EUDAMED, CIV-11-09-002381). It consists of a handheld, battery driven electrical stimulator connected to an ear electrode placed in contact with the skin of the concha. Impedance is measured automatically and insufficient electrode contact with the skin evokes an alarm. During stimulation, series of electrical pulses (pulse width: 250 μs, frequency: 1 Hz or 25 Hz, duty cycle: 30s on, 30 s off, to avoid habituation) are applied to the skin of the concha. Stimulus intensity was individually fitted during visit 2 to elicit a tingling but not painful sensation, and could later be adjusted by the patient as needed. Patients were asked to stimulate for a total of 4 h per day (in sessions of 1 to 4 h, a specific distribution over the day or interval between sessions was not required), and were free to stimulate for an additional hour if they thought this was useful, e.g. for treatment of acute headache. The effect of such acute treatment was not recorded. Stimulation parameters of the 25 Hz group were chosen so that with 4 h of daily stimulation, the number of electrical stimuli per day would be similar to those normally used for invasive vagal nerve stimulation in patients with epilepsy. The 1 Hz stimulation was intended as an active control. The active control was chosen in order to avoid un-blinding of the subjects.

### Primary and secondary outcome parameters

All outcome measures refer to change from baseline (the 4-week period between visits 1 and 2) to the evaluation period (the 4-week period between visits 5 and 6, Fig. 1). The primary outcome measure was mean change in *headache days per 28 days*. A headache day was defined as a calendar day with headache of ≥ 4 h duration or headache successfully aborted by acute headache medication or any other treatment known to be typically effective in the specific patient (e.g. sleep, progressive relaxation exercises).

Secondary outcome parameters were: (1) percentage of “responders” (subjects having at least 50 % reduction of headache days per 28 days from baseline to evaluation); (2) change in mean headache intensity on days with headache; (3) change in days with acute headache medication intake per 28 days; (4) change in headache-related disability, as assessed by the MIDAS and HIT-6 questionnaires; (5) number and type of adverse events.

### Statistical analysis

Mean ± standard deviation (SD) is reported unless stated otherwise. The threshold for significance of statistical comparisons was set at p < 0.05. Statistical analysis was performed both on ITT and on per protocol basis (PP). For the ITT analysis, a last observation carried forward approach was used for patients who dropped out during the course of the study.

Group comparisons at baseline, of duration of the treatment period, compliance or number of patients affected by adverse events were done using Mann–Whitney *U*-Test or Fisher’s exact test as appropriate. Analysis of the primary endpoint was done using an analysis of covariance (ANCOVA) model with the factors treatment group (1 Hz vs. 25 Hz) and sex as categorical variables and baseline values as covariate. The same type of ANCOVA was used for the analysis of the following secondary outcome parameters: change in mean headache intensity, change in days with acute headache medication intake per 28 days and change in MIDAS and HIT-6 scores. The number of responders was compared between groups using a logistic regression model that included treatment group and sex as factor and the number of headache days per 28 days at baseline as covariate. An estimate of the treatment odds ratio (Wald method) was derived from this model.

## Results

**Fig. 3:**
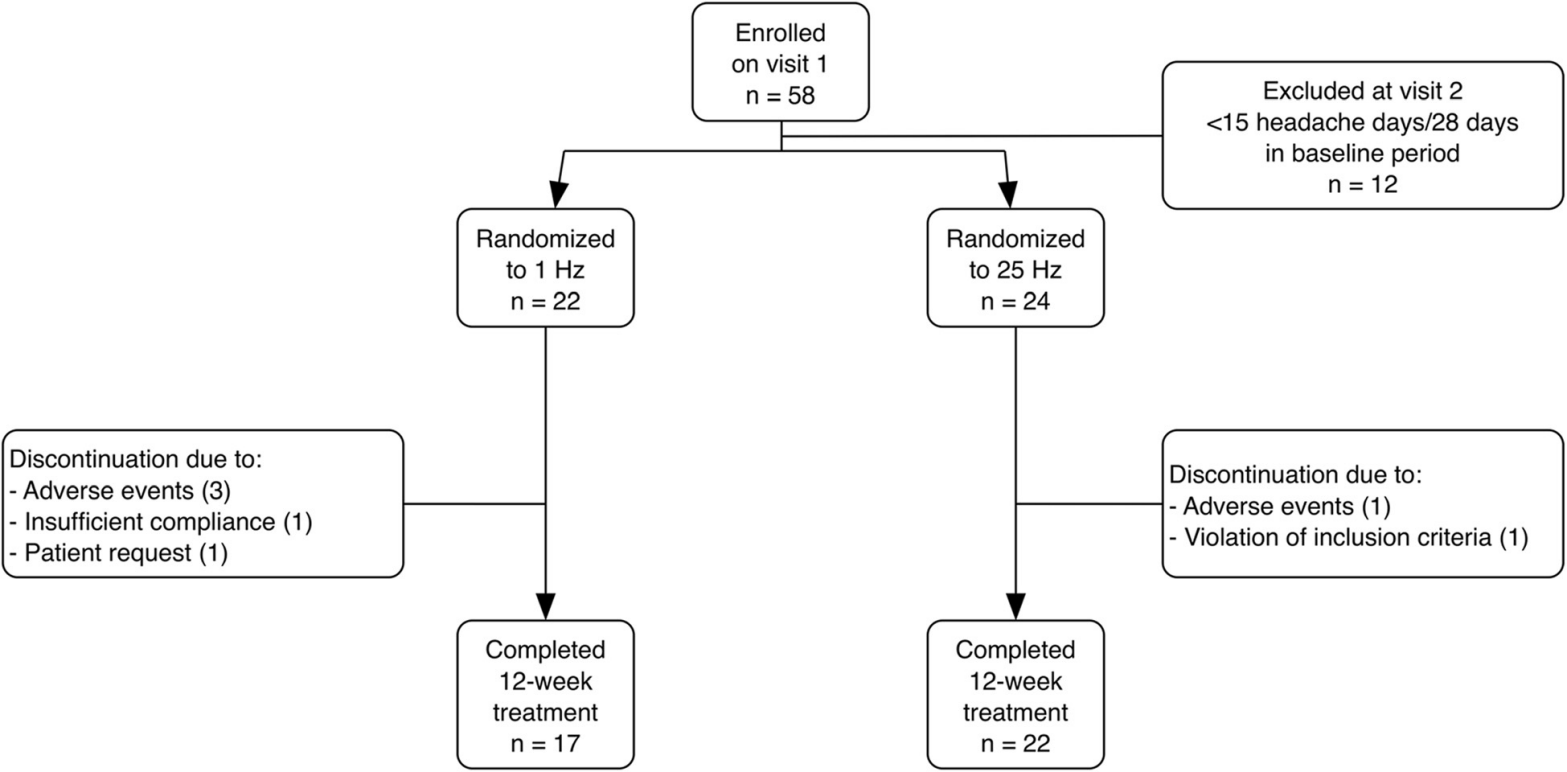
Patient disposition

The study was conducted between March 2012 and July 2014. A total of 46 patients were randomized to the 1 Hz group (*n* = 22) or the 25 Hz group (*n* = 24, ITT). 6 patients dropped out during the study. Reasons for dropouts were: adverse events in 4 patients (treatment-related stimulation site ulcer in 3 patients, gastrectomy not related to treatment in 1 patient), insufficient compliance in 1 patient, patient’s request in 1 patient. One additional patient was excluded from the per protocol (PP) analysis after the end of the study because of violation of inclusion criteria (<15 headache days per 28 days in the screening period). This left 17 patients in the 1 Hz group and 22 patients in the 25 Hz group for the PP analysis (Fig. 3) Demographic and headache characteristics of the population are shown in Table 1. There were no significant differences between both groups.

**Table 1:** Baseline characteristics of the cohort

	Intention-to-treat analysis			Per protocol analysis		
	1 Hz (*n* = 22)	25 Hz (*n* = 24)	Group comparison	1 Hz (*n* = 17)	25 Hz (*n* = 22)	Group comparison
Age	43.8 ± 11.5	39.3 ± 12.4	*p* = 0.21	44.1 ± 11.4	39.0 ± 12.5	*p* = 0.21
Females	18	21	*p* = 0.69	13	19	*p* = 0.68
Headache days/28 days	19.4 ± 4.0	18.9 ± 5.1	*p* = 0.47	19.1 ± 3.7	19.2 ± 4.7	*p* = 0.66
Headache intensity (NRS: 0–10)	5.2 ± 1.5	5.0 ± 1.5	*p* = 0.73	5.0 ± 1.5	5.0 ± 1.5	*p* = 0.98
Migraine history (years)	27.1 ± 13.0	20.4 ± 12.1	*p* = 0.08	27.8 ± 11.5	21.4 ± 12.1	*p* = 0.11
Days with acute headache medication/28 days	10.3 ± 6.4	8.2 ± 4.9	*p* = 0.24	11.1 ± 6.6	8.6 ± 4.8	*p* = 0.17
MIDAS score	76.8 ± 64.8	83.6 ± 56.0	*p* = 0.55	77.2 ± 70.1	82.1 ± 58.0	*p* = 0.71
HIT-6 score	64.3 ± 4.7	66.0 ± 4.1	*p* = 0.25	64.8 ± 5.0	66.0 ± 4.2	*p* = 0.55
BDI	6.9 ± 5.7	7.9 ± 5.6	*p* = 0.59	6.9 ± 5.9	7.2 ± 5.0	*p* = 0.95

Demographic and headache characteristics assessed at the first visit or during the baseline period (4 weeks) are given. Values are mean ± SD or numbers of subjects. Results of Mann–Whitney *U* test or Fisher’s Exact test are given. Headache intensity (NRS: numerical rating scale 0–10)

*MIDAS* migraine disability assessment, *HIT* headache impact test, *BDI* beck’s depression inventory

### Primary outcome measure

**Fig. 4:**
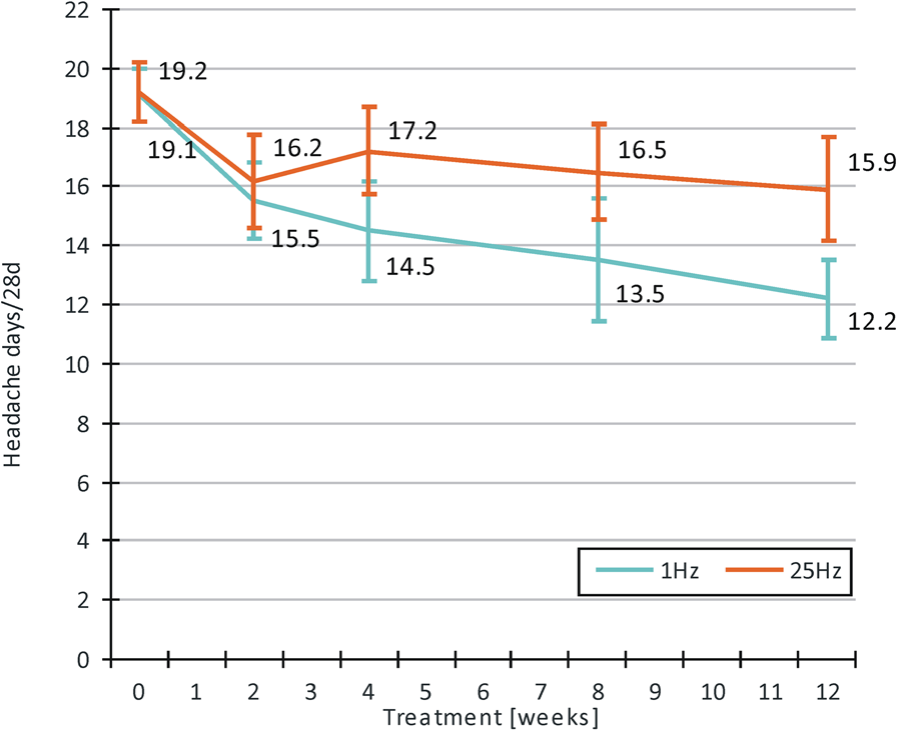
Mean course of number of headache days per 28 days during t-VNS treatment. Results of the per protocol set are shown (1 Hz: *n* = 17, 25 Hz: *n* = 22). Values are mean ± SEM. Mean values are also given in the figure

PP-analysis indicated a significant decrease in headache days per 28 days from baseline to evaluation, which was significantly larger in the 1 Hz group than in the 25 Hz group (F[35] = 4.82, *p* = 0.035, Table 2). In the 1 Hz group, the reduction amounted to −7.0 days per 28 days (36.4 % reduction from baseline), while the 25 Hz group reached only −3.3 days (17.4 % reduction from baseline). In the ITT analysis, there also was a significant decrease in headache days per 28 days in both groups, but no significant group difference (F[42] = 2.94, *p* = 0.094, Table 2). Visual inspection of headache days per 28 days over the treatment period revealed a continuous decrease in the 1 Hz group, while a steady state was reached after 14 days in the 25 Hz group (Fig. 4).

**Table 2:** Results of primary and secondary treatment outcome measures

	Intention-t-treat analysis			Per protocol analysis		
	1 Hz (*n* = 22)	25 Hz (*n* = 24)	Group comparison	1 Hz (*n* = 17)	25 Hz (*n* = 22)	Group comparison
Change in headache days/28 days	**−5.6 ± 5.0**	**−3.0 ± 5.3**	F[42] = 2.94	**−7.0 ± 4.6**	**−3.3 ± 5.4**	**F[35] = 4.82**
	**(−5.9; −0.5)**	**(−8.5; −3.2)**	*p* = 0.094	**(−9.6; −4.1)**	**(−5.9; −0.4)**	***p*** **= 0.035**
Responder (50 % reduction in headache days)	5 (22.7 %)	3 (12.5 %)	*OR* = 2.44	5 (29.4 %)	3 (13.6 %)	*OR* = 3.21
			*p* = 0.29			*p* = 0.18
Change in headache intensity (NRS 0 – 10)	−0.1 ± 1.1 (*n* = 20)	0.2 ± 1.0	F[40] = 0.30	0.02 ± 1.2 (*n* = 15)	0.2 ± 1.0	F[33] = 0.28
	(−0.2; 0.9)	(−0.4; 0.7)	*p* = 0.58	(−0.4; 0.8)	(−0.2; 0.9)	*p* = 0.60
Change in days with acute headache medication in 28 days	**−2.0 ± 4.2**	**−1.3 ± 4.4**	F[42] = 0.01	**−2.7 ± 4.5**	**−1.6 ± 4.1**	F[35] < 0.01
	**(−4.2; −0.3)**	**(−4.4; −0.3)**	*p* = 0.91	**(−4.7; −0.4)**	**(−4.7; −0.3)**	*p* = 0.96
Change in MIDAS score	**−18.7 ± 28.0**	**−21.8 ± 54.5**	F[42] < 0.01	**−24.2 ± 29.8**	**−26.5 ± 53.9**	F[35] < 0.01
	**(−38.6; −0.9)**	**(−39.2; −0.8)**	*p* = 0.98	**(−43.2; −4.0)**	**(−42.1; −3.7)**	*p* = 0.96
Change in HIT-6 score	**−2.5 ± 6.8**	**−3.8 ± 5.5**	F[42] = 0.12	**−3.8 ± 7.1**	**−3.9 ± 5.69**	F[35] = 0.01
	**(−6.7; −0.7)**	**(−7.3; −1.2)**	*p* = 0.73	**(−7.8; −1.1)**	**(−7.6; −1.0)**	*p* = 0.93

Change refers to change from the 4-week baseline period to the last 4 weeks of the 12-week treatment period. Means, SDs and 95 % confidence intervals are given. For the responder analysis, numbers of subjects and percent of the total group are given. Significant differences are marked in bold. Number of subjects is given in parentheses, where different from the total group. Primary outcome parameter: change in headache days/28 days

*MIDAS* migraine disability assessment, *HIT* headache impact test, *NRS* numerical rating scale 0–10

### Secondary outcome measures

Results of secondary outcome measures and the corresponding statistics are summarized in Table 2. The number of responders (>50 % improvement in headache days) was in the 1 Hz group (PP) 29.4 % and in the 25 Hz group (PP) 13.6 %. Headache intensity was not significantly changed by t-VNS in either treatment group, and there were no group differences. The number of days with intake of acute headache medication as well as the MIDAS and HIT-6 scores were significantly reduced in both treatment groups, there were no group differences.

### Treatment duration and compliance

Results and statistics are listed in Table 3. Duration of the treatment period was similar between groups. The average number of stimulated hours per day during the treatment period was around 3.4 in all groups, corresponding to around 85 % of the requested 4 h of daily stimulation, indicating good compliance with treatment. There were no significant group differences.

**Table 3:** Duration of treatment period and compliance with stimulation during the treatment period

	Intention-to-treat analysis			Per protocol analysis		
	1 Hz (*n* = 22)	25 Hz (*n* = 24)	Group comparison	1 Hz (*n* = 17)	25 Hz (*n* = 23)	Group comparison
Treatment period (days)	77.9 ± 25.8	85.7 ± 11.4	*p* = 0.22	89.0 ± 8.4	87.5 ± 7.5	*p* = 0.67
Average number of stimulated hours per day	3.42 ± 0.59	3.44 ± 0.61	*p* = 0.69	3.34 ± 0.62	3.44 ± 0.62	*p* = 0.51

Mean ± SD values are given. Treatment period indicates the number of days between visits 2 and 6. The average number of stimulated hours per day of the treatment period is given. Patients were requested to stimulate 4 h per day during the treatment period. The real average stimulation time per day was slightly lower

### Safety and tolerability

Adverse events (AEs) were analysed in the full analysis set (safety set) and summarized in Table 4. The number of treatment emergent AEs (AEs occurring after initiation of treatment) was higher in the 25 Hz group (112 events, 76 treatment-related events) as compared to the 1 Hz group (67 events, 39 treatment-related events, Table 4),. Most AEs were mild or moderate in severity and resolved without sequelae. The most frequent treatment-related AE were local problems at the stimulation site, such as mild or moderate pain, paresthesia, or pruritus during or after stimulation, and erythema, ulcer or scab (31 events in 10 patients in the 1 Hz group, 70 events in 17 patients in the 25 Hz group, *p* = 0.14). Treatment-related AEs leading to discontinuation of the study were stimulation site ulcer (accompanied by pain, paresthesia, or pruritus) in 2 patients of the 1 Hz group and in 1 patient of the 25 Hz group. These three cases of application site ulcer occurred early during the study. After that, patients were asked to specially care for the skin of their ear after each use of the NEMOS® device, using a custom rich skin cream, and no more cases of application site ulcer occurred. There were no treatment-related SAEs. Three SAEs, leading to hospitalization of the patient, were recorded during the whole study (infectious mononucleosis, gastrectomy, intervertebral disc protrusion).

**Table 4:** Overview of adverse events (safety set)

	1 Hz (*n* = 22)		25 Hz (*n* = 24)	
	Number of events	Number of patients (%)	Number of events	Number of patients (%)
Treatment emergent AEs	67	17 (77.3 %)	112	19 (79.2 %)
Treatment-related AEs	39	11 (50.0 %)	76	17 (70.8 %)
Stimulation site treatment-related TEAEs	31	10 (45.5 %)	70	17 (70.8 %)
All serious AEs (including pre-treatment SAEs)	2	2 (9.1 %)	0	0
Serious treatment emergent AEs	2	2 (9.1 %)	0	0
Serious treatment-related AEs	0	0	0	0
Treatment-related AEs leading to discontinuation of study	8	4 (18.2 %)	4	1 (4.2 %)
Death	0	0	0	0

## Discussion

The present monocentric, randomized, controlled, double-blind, parallel-group clinical trial provides evidence that daily treatment with auricular t-VNS is effective in chronic migraine.

Both in the 1 Hz and the 25 Hz group the number of headache days per 28 days decreased significantly by 7.0 and 3.3 days, respectively (PP-analysis, Table 2), with a significantly larger reduction in the 1 Hz compared to the 25 Hz group (*p* = 0.035). 29.4 % of the patients in the 1 Hz group and 13.6 % of the patients in the 25 Hz group achieved a reduction of more than 50 % in headache days (“responder”). With an absolute reduction in headache days per 28 days by 7.0 in the 1 Hz group and a mean group difference of 2.7 headache days, the effect of auricular t-VNS was comparable to the effects of topiramate and onabotulinumtoxin A versus placebo. Previous trials in chronic migraine with topiramate for 4 months have shown a reduction in headache days per month of 3.5 and 6.4 days in the verum group, which exceeded the effect in the placebo group by 3.7 and 1.7 days, respectively [6, 30]. In the large PREEMPT trials onabotulinumtoxin A was able to reduce the number of headache days per month in chronic migraine patients by 9.0 and 7.8 days after 6 months, which exceeded the placebo effect by 2.3 and 1.4 days, respectively [5, 31]. Compared to previous trials investigating neurostimulation devices the results are favorable. In the ONS trials for chronic migraine, reduction of headache days after 3 months was by 6.7, 5.5 and 6.1 days in the verum group, which exceeded the sham group by 5.2, 1.6 and 3.1 days, respectively [8–10]. However, none of these studies reached significance for its primary end point. Transcutaneous supraorbital neurostimulation has so far only been tested in episodic migraine, achieving a reduction by 2.5 headache days from a baseline of 7.8 headache days, which was 2.3 days more than placebo [11].

It has to be mentioned that the study was planned as a trial with an active comparator in order to be sure that the patients were blinded and that we expected that the 25 Hz stimulation would be more effective than the 1 Hz stimulation, corresponding to the results from the use of invasive VNS in epilepsy [32, 33]. This means that it is very unlikely that partial unblinding may have affected the results, as the local sensation is more intense with 25 Hz stimulation, and the study physicians expected the 25 Hz stimulation to be more effective. However, it is not clear why the 1 Hz stimulation was more effective than the 25 Hz stimulation. The mechanisms by which VNS influences chronic migraine may be different from those in epilepsy. In addition, activation of central nervous system structures by stimulation of thickly myelinated sensory fibers in the auricular branch of the vagus nerve may require different stimulation patterns than the cervical branch, which is a mixed nerve with myelinated and non-myelinated efferent as well as afferent fibers. As no dose–response or frequency-response data are available for any neurostimulation method in migraine treatment, the question whether frequency or total number of stimuli influence the result remains open.

Analgesic effects of electrical low-frequency stimulation (LFS) in various pain models have been demonstrated in man and rodents [34]. Electrical pulse series with optimum frequency of 1 Hz for 20 min significantly suppressed nociceptive signaling and pain perception by approximately 40 % for hours [35, 36]. This phenomenon of long-term depression (LTD) has been shown in the spinal system [37–41] and in the craniofacial area [42–44]. Stimulation parameter of t-VNS in the present study resemble electrical LFS and could have provoked LTD of nociceptive processing in the spinal trigeminal nucleus that plays a critical role in migraine pain [45]. Actually, the auriculotemporal nerve, a branch of the trigeminal nerve, supplies the outer ear and could, therefore, mediate access of electrically evoked neural signals to brainstem nuclei of the trigeminal nerve [46]. Thus, LTD could be a mechanism that might, at least, contribute to the analgesic effect of t-VNS in the present study.

In fact, other stimulation parameters might be even more effective than the 1 Hz stimulation, and the 25 Hz stimulation might have been partially active in the present study, possibly reducing the effect in the group comparison. Indeed, 25–30 Hz stimulation has been shown to significantly reduce experimental pain in humans [23] and seizures in rodents [18]. In addition, in the present study both groups significantly improved in headache-related disability measures (MIDAS and HIT-6), and reduced their intake of acute headache medication, although it cannot be determined if this is due to the placebo effect or due to stimulation effects in both groups. The missing significant difference in the reduction of the MIDAS and HIT6 between the 1Hz and the 25Hz group is probably due to the too small sensitivity of these tests in detecting differences in quality of life. Furthermore, it is unclear if 25 Hz stimulation also have a mood stabilizing effect which influences the ratings in the used tests.

Furthermore, it is still not clear how vagus nerve stimulation interferes with migraine generation. One possibility is a direct or indirect inhibition of nociceptive trigeminal neurons by vagal activation. Indeed, animal data show that afferent vagal stimulation can reduce the activation of nociceptive neurons in the caudal trigeminal nucleus in response to noxious stimulation of the face or dura [47–49]. This might be due to the existence of dense reciprocal connections between the spinal trigeminal nucleus and the nucleus tractus solitarii (NTS) which is the major target of vagal afferents [50]. Responses of spinal trigeminal neurons might also be reduced by activation of the descending pain inhibitory systems. Although this has not been shown directly for the trigeminal area, animal studies showed that vagal nerve stimulation can activate descending pain inhibitory systems, probably involving projections from the NTS to the nucleus raphe magnus and the locus coeruleus, which are at the origin of serotonergic and noradrenergic descending pain inhibitory pathways [51]. Alternatively, VNS might exert migraine prophylactic actions by modifying cortical excitability. Altered cortical excitability in chronic migraine has been demonstrated in various electrophysiological measurements is thought to contribute to its pathogenesis [52]. Several lines of evidence indicate that the cortical excitability is increased in chronic migraine patients: 1) There is a reduced habituation of the blink reflex interictally [53]. 2) The magnetic suppression of perceptual accuracy was decreased in patients with chronic migraine compared to episodic migraine and controls which may indicate also a higher cortical excitability [54]. 3) Analysis of the high frequency somatosensory evoked potentials showed early response sensitization and late habituation, most probably due an increased coupling between thalamus and cortex in chronic migraine [55]. Afferent vagal information is relayed via the NTS and the parabrachial nucleus to several subcortical and cortical regions, including thalamus, insula and lateral prefrontal cortex. In addition, the NTS has strong projections to the locus coeruleus and the nucleus raphe magnus which provide widespread noradrenergic and serotonergic innervation of the cortex [56]. Modulation of cortical excitability via these pathways is thought to be important for the anticonvulsant effects of VNS [33]. Increased GABA levels have been found in the cerebrospinal fluid of epilepsy patients treated with VNS, suggesting an increase in inhibitory neurotransmission [57]. Auricular t-VNS increases parasympathetic activity and/or reduces sympathetic activity [19], which might also affect cortical excitability, maybe by mechanisms similar to those assumed for the migraine preventive effects of beta-blocking agents [58]. In summary, VNS is well positioned to alter cortical excitability, especially to reduce cortical hyperexcitability. Direct evidence that this interferes with pain processing or migraine generation is currently lacking. It would be interesting to repeat the above described experiments which showed an increased cortical excitability in chronic migraine under t-VNS stimulation. A third possibility is that the anti-migraine action of VNS relies on modification of transmitter release from efferent parasympathetic fibers innervating dural vessels, e.g. fibers stemming from the spheno-palatine ganglion. The release of neurotransmitters, especially calcitonin-gene related peptide (CGRP), at dural vessels with subsequent neurogenic inflammation and sensitization of primary afferents is thought to play an important role in migraine pathophysiology [59]. Parasympathetic fibres innervating the dura mater release vasoactive intestinal polypeptide (VIP) and pituitary adenylate cyclase-activating polypeptide (PACAP), which are potent vasodilatators and thought to contribute to sensitization of nociceptive trigeminal primary afferents. Increased peripheral blood VIP levels have been detected in chronic migraine [60], and intravenous administration of PACAP has been shown to induce migrainous headache in migraine patients [61], suggesting that both transmitters are related to migraine pathophysiology. Although auricular t-VNS stimulates only vagal afferents, there are close connections between afferent and efferent parasympathetic brainstem centers, making an influence of VNS on dural efferents likely.

A major practical advantage of auricular t-VNS is good tolerability and safety. For comparison, in the pooled topiramate trial analysis, 1 out of 4 patients (25 %) dropped out because of intolerable adverse effects [62]. In our study only 3 of 46 patients (7 %) dropped out due to side effects of t-VNS. All three cases occurred early in the study and were due to stimulation site ulcer which later in the study could be prevented by appropriate skin care. Another advantage of t-VNS therapy is that it can be combined with any other drug treatment without risking cumulative adverse effects or pharmacodynamic interactions. In addition, auricular t-VNS allows patients to continue routine activities, leading to a high compliance with stimulation times (around 85 % on average). However, long-term effects and sustainability of efficacy of t-VNS are still unknown and need to be demonstrated in appropriate open-label trials.

## Conclusions

In conclusion, the present parallel-group randomized controlled trial, provides evidence that auricular t-VNS at 1 Hz for 4 h daily is effective for chronic migraine prevention over 3 months. The absolute reduction in headache days (7.0) and the difference between groups (2.7 headache days) is comparable to the effects of topiramate and onabotulinum toxin A in chronic migraine prevention. The t-VNS treatment also results in a meaningful improvement in the quality of life as assessed by MIDAS and HIT 6. The safety profile was favourable and compliance with daily stimulation was high.
